# Polyurethane Recycling: Conversion of Carbamates—Catalysis, Side-Reactions and Mole Balance

**DOI:** 10.3390/polym14224869

**Published:** 2022-11-11

**Authors:** Shahab Zamani, Jean-Paul Lange, Sascha R. A. Kersten, M. Pilar Ruiz

**Affiliations:** 1 Sustainable Process Technology, Faculty of Science and Technology, University of Twente, Drienerlolaan 5, 7522 NB Enschede, The Netherlands; 2 Shell Global Solutions International B.V., Shell Technology Centre Amsterdam, Grasweg 31, 1031 HW Amsterdam, The Netherlands

**Keywords:** polyurethanes, recycling, carbamates, isocyanates

## Abstract

Diisocyanates, a key monomer in polyurethane, are generally lost during recycling. Polyurethane alcoholysis to carbamate and subsequent cracking to isocyanate represents a promising, phosgene-free recycling route. This work reports the thermal and catalytic cracking of a model carbamate (Methyl N-phenyl carbamate, MPC) to isocyanate (Phenyl isocyanate). Multiple catalysts (ZnO, Bi_2_O_3_, Al_2_O_3_, and Montmorillonite K-10) were evaluated in a closed system (batch autoclaves) to decompose MPC at temperatures of 160–200 °C, with a thorough analysis of the products and high (≥90%) mole balance. The thermal reaction was very limited at these temperatures, whereas the catalytic reaction led mainly to aniline and urea and seemed to be dominated by water adsorbed on the catalyst surface.

## 1. Introduction

Polyurethanes, a versatile family of polymers, were discovered by Otto Bayer in 1937 [1] and are mainly used as flexible foams in mattresses and, to a lesser extent, as rigid foams for insulation. Interest in novel PU applications has grown recently, such as the use of superhydrophobic polyurethane to cure oil spills [2]. They are a major thermoset polymer used worldwide, with an annual global production of more than 23 million tons in 2020 [3,4].

**Scheme 1:**
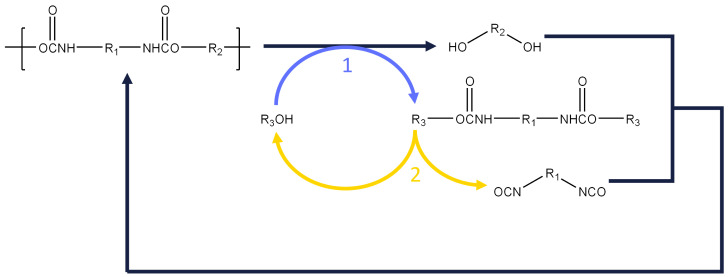
Recycling pathway of the polyurethanes toward the production of isocyanates and polyols (1): alcoholysis; (2) thermal cracking of carbamates. R_1_,R_2_,R_3_ are alkyl groups.

We discuss here opportunities and challenges in depolymerizing polyurethanes (PUR) to their constituting monomers, a polyol and an isocyanate (diisocyanate), as illustrated in Scheme 1 and detailed below.

Commonly, the isocyanates are toluene diisocyanate (TDI) and methyl diphenyl diisocyanate (MDI), with 1,6-hexamethylene diisocyanate (HDI) being occasionally used as well [5,6,7]. Polyols are often polyethers made by oligomerizing propylene oxide onto glycerol. Indeed, the growing demand for sustainability and the ever-increasing environmental concerns about waste accumulation necessitates proper recycling and disposal solutions for polymers. While considerable efforts have been made to identify more ecologically friendly ways to produce PUR, it does not address the challenge of reclaiming the discarded and used PUR [8,9,10].

Over the years, many alternatives have been investigated to aid in the recycling of existing polyurethane and assist in its sustainable production [6,9,10,11,12,13,14]. The best course of action for PUR post-use is reusing. While reusing sounds promising, it would be impractical to apply it to foam mattresses for hygiene reasons. Hence, the most common approach is mechanical downcycling, e.g., to pillows after crushing, shredding, etc.

Incineration is another common method used to handle waste PUR. However, the environmental concerns associated with incineration and the loss of valuable products make this approach undesirable. Therefore, PUR’s chemical and thermochemical recycling could be a valuable route for recycling. PUR should be amenable to depolymerization, in principle, based on thermochemical considerations [15]. Over the decades, multiple researchers have investigated the decomposition of PUR with different methods, such as glycolysis [10,13,14]. Pyrolysis could also break PUR into smaller, though less valuable, molecules.

Glycolysis/alcoholysis, specifically split-phase glycolysis [14], has been proven to be a suitable method for recovering polyols [10,13,14,16,17]. However, the recovery of the isocyanate components appeared more complicated and has yet to be developed to the same extent as the polyol recovery. The isocyanate phase contains carbamates, amines, and possibly some organic contaminations alongside the glycol used in the glycolysis step.

Thermal and catalytical cleavage of carbamates to valuable products has been investigated. However, the primary focus has been on the recovery of amines, which can be converted to isocyanate via phosgenation [10,11,12,13,14,18,19,20].

**Scheme 2:**
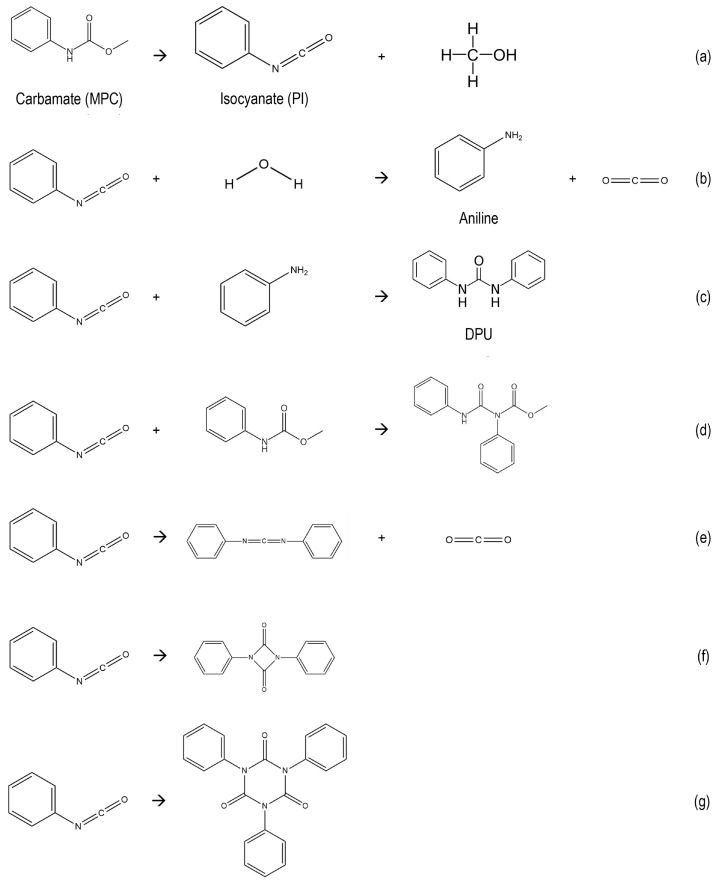
Possible route of reaction of isocyanates in a carbamate thermal cracking process.

As an alternative, the thermal cleavage of carbamates could produce isocyanates, which would be a high-value recycling pathway. The decomposition of carbamates is highly endothermic; it must, therefore, be operated at high temperatures, preferably above 150 °C [7,21,22,23]. However, numerous undesired side-reactions can be envisaged. Hence, to produce and retain isocyanates from the thermal cleaved carbamates, it is essential to understand the possible side reactions that the isocyanates can go through. To facilitate this discussion, we will consider methyl-phenyl carbamate as a model component for aromatic dicarbamate and investigate its desired reaction (Scheme 2, reaction a) and presumed undesired side-reactions (Scheme 2, reactions b–g).

Isocyanates could react with water (Scheme 2, reaction b), in which an amine (e.g., aniline) is produced. Then, the formed amine could further react with isocyanates to form substituted urea (e.g., diphenyl urea, Scheme 2, reaction c). Along with these reactions, isocyanates could also react with unconverted carbamate (Scheme 2, reaction d) or with themselves to form dimers and trimers (e.g., Scheme 2, reaction e–g) [7].

Over the decades, few studies have investigated the decomposition of different carbamates to isocyanates, generally intending to find better catalysts for operation at a lower temperature [22,24]. Many studies report reasonable to excellent selectivities (80%) but do not provide mass or mole balances nor discuss the possible formation of heavy products expected from Scheme 2d–g. They also apply batch conditions with high catalyst/carbamate ratios, up to 1:1 by weight, which could favor the adsorption of heavy products inside the pores of the catalyst.

For instance, Uriz et al. [25] have evaluated the decomposition of multiple carbamates. They reported a high conversion for the 4,4${}^{\prime }$-methylenebis-(N-carbomethoxianiline) and 2,4-toluene-bis(N-carbomethoxyurethane). However, their experiments use a large amount (1:1 wt basis) of catalysts (a montmorillonite MMK); the selectivities are reported based on the identified products, which are limited to the mono and diisocyanate that form only, and information about mole balances is not reported.

Similarly, a very high yield (90% mole) for TDI is reported in the work of Wang et al. [26] within two hours with ZnO and aluminium powder as catalysts. Wang et al. [27] also evaluated different metal oxide catalysts for the decomposition of 4,4${}^{\prime }$-methylenebis-(N-carbomethoxianiline), where the highest selectivity that they obtained for MDI was 13% for the conversion of 63.6%. However, no mole balance is provided.

A noteworthy exception is the study of Lewandowski et al. [28] that was carried out under flow conditions (5 wt% feed, 8 g/${\mathrm{dm}}^{3}$ min, reaction time 80–110 min) in the absence of catalyst and that reports mole balances over 80%. Their product consisted mainly of the desired isocyanate, even at high conversions. This is a proof of concept for the purely thermal decomposition of the carbamates toward isocyanates without using any catalysts.

Despite the potential of this alternative to produce isocyanates, the reported literature is scarce and does not provide general agreement on the detailed chemistry and side reactions, the role of the catalysts, or the desired experimental conditions. This scenario encouraged us to revisit this reaction and carefully investigate the reaction scheme, the by-products and the mole balances. First, a reliable analysis method was developed to identify and quantify the reactants and primary products involved in the system. Later, the role of different catalysts and operating conditions were experimentally tested to identify the key parameters affecting the production of isocyanates. These results will help advance on the chemical recycling of polyurethanes by allowing the development of integrated processes for the recovery of both isocyanates and polyols.

## 2. Materials and Methods

Methyl N-phenyl carbamate (CAS: 2603-10-3) was purchased from TCI chemicals. Phenyl isocyanate (≥98%), aniline (≥99%), 1,3-Diphenylurea (98%), 1-(2-Pyridyl) piperazine (≥99%), Dimethyl sulfoxide (anhydrous ≥ 99.9%) and diphenyl ether (≥99%) were purchased from Sigma-Aldrich.

Acetonitrile (HPLC grade), water (HPLC grade), ammonium acetate (≥98%), and formic acid (For LC-MS 98–100%) were all purchased from Sigma-Aldrich for use in analytical equipment (LC).

Catalysts include montmorillonite K10 (H_2_Al_2_(SiO_3_)_4_−nH_2_O, surface area 220–270 m^2^/g), zinc oxide (nanopowder, ≤100 nm, 10–25 m^2^/g), and aluminium oxide (50–300 mesh, acid, 155 m^2^/g) purchased from Sigma-Aldrich and Bismuth(III) oxide (nanopowder 99.9%, 3.8–5.5 m^2^/g) was purchased from Fischer scientific. The catalysts were stored in an oven (110 °C) prior to experiments.

### 2.1. Experimental Procedure

Methyl N-phenyl carbamate (MPC) was selected as the model component for these experiments, as it is the simplest aromatic carbamate.

**Figure A1:**
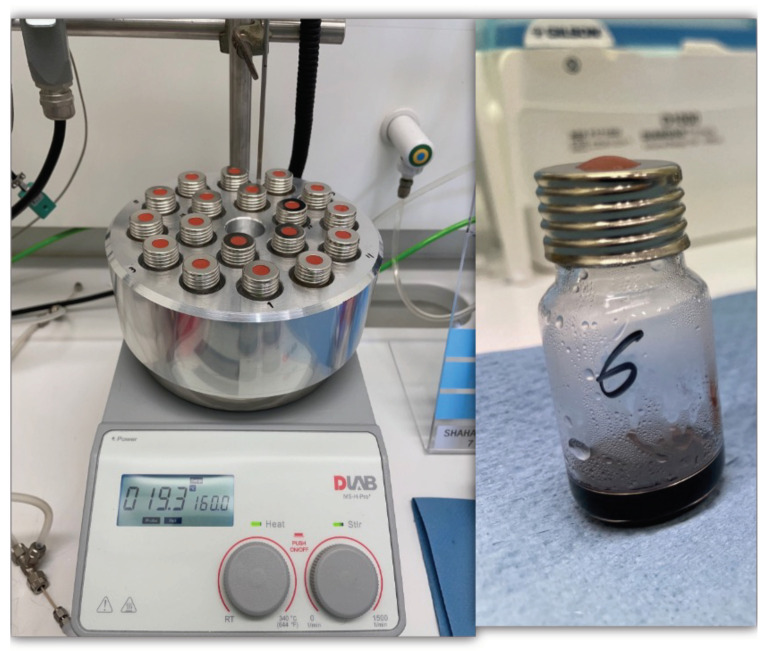
Autoclave heating block and setup were used in these experiments.

The catalytic thermolysis of MPC was carried out in 6 mL glass autoclaves (Duran^®^ culture tube GL14) that were stirred with a small magnetic bar, closed with septa and placed in an aluminium block shown in Figure A1. The aluminium block was built in-house and placed on a hot plate to heat the autoclaves to the desired reaction temperature.

The autoclaves containing catalysts were charged with 1 mL of a stock mixture consisting of the solvent (diphenyl ether) and a known quantity of the MPC (typically 8 wt%). No catalyst was added to the system for a purely thermal experiment. The amount of catalyst was calculated as the ratio of the catalyst mass to the MPC within the autoclave.

**Figure A2:**
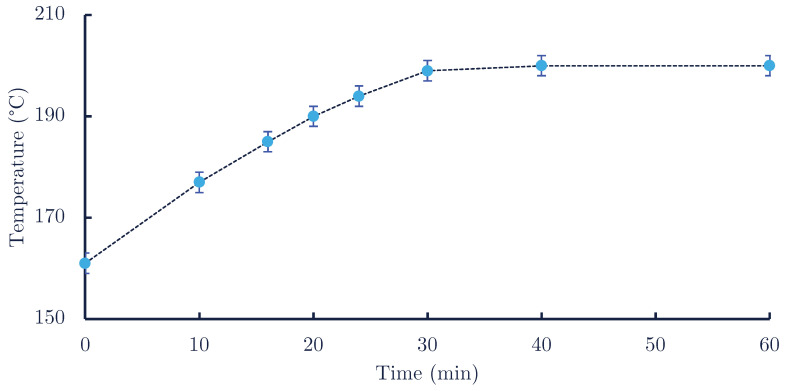
Temperature profile during heating in the experiments, assuming starting time to be once the block reaches 160 °C.

The reaction temperature for the experiments was usually 180–200 °C. The beginning of the experiment was defined as when the autoclave temperature surpassed 160 °C. It usually took about 30 min for the block to reach 200 °C, assuming 160 °C as the starting point. Therefore, samples taken at an earlier time were at lower temperatures. The temperature profile is presented in Figure A2.

Once the desired reaction time was completed, the autoclaves were removed from the reaction block and placed in the open air for 10 min to cool the reaction mixture. Once the mixture was cooled, the autoclave was opened, a sample was extracted via a syringe, and the mixture was immediately stabilized by derivatization to stop any further reaction. The derivatization step is further discussed in the Section 2.2.

The derivatized samples were then treated and analyzed using liquid chromatography (LC) with a UV detector. The reported carbamate conversion (*X_A_*) is based on the reacted mole of the feedstock to the moles fed to the system (Equation (1)), which were quantified by comparing it to the corresponding calibration line.

(1) $$X_{A}=\frac{mole_{fed}-mole_{out}}{mole_{fed}}*100$$

(2) $$Y_{Molar\;yield}=\frac{mole_{product\;formed}*stoichiometric\;coefficient}{mole_{feed}}$$

The yield for the diphenyl urea (DPU) is calculated considering that 2 moles of MPC are needed to make 1 mole of DPU (Equation (2)). Mole balances were based on known compounds (MPC equivalent). Therefore, the amounts of MPC, phenyl isocyanate, diphenyl urea, and aniline were measured in the analysis method. Any missing (≤100%) mole balance could be related to aromatic compounds, which were not identified. Great efforts were made to close the mole balance to ≥90 mol% over a broad conversion window, as discussed in detail below.

### 2.2. Analytical Method

Different analytical methods were evaluated to establish a reliable method for measuring isocyanates. Due to their high reactivity and thermal degradation of the carbamates at temperatures higher than 160 °C, analytical techniques such as gas chromatography were not reasonable. While some previous researchers have worked on gas chromatography methods [29,30,31,32], we did not find them reliable for the accurate measurements of isocyanates and thermally unstable carbamates due to the possibility of carbamate decomposition taking place within the analysis machine and the toxicity associated with airborne isocyanates.

#### 2.2.1. Sample Preparation

**Scheme 3:**
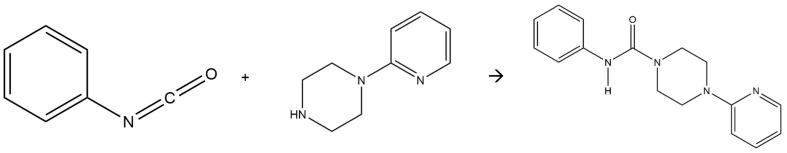
Derivatisation of phenyl isocyanates via reaction with PP.

One of the main challenges in analyzing isocyanates is their high reactivity and toxicity. To make these compounds stable and safe for analysis, they needed to be neutralized by a derivatization reaction of the isocyanate into a urea derivative. For this purpose, an amine equivalent of isocyanates is commonly measured and quantified to find the quantity of isocyanates. Usually, this is conducted by reacting the isocyanate with an amine such as dibutyl amine [7,33,34]. This work used 1-(2-pyridyl) piperazine (PP) as the derivatization agent [35,36,37]. Scheme 3 shows the reaction of PI with PP.

A derivatization solution was prepared in which PP was mixed with dimethyl sulfoxide (DMSO) in stoichiometric excess to isocyanates (phenyl isocyanate or its precursor MPC in this case, 10% molar excess to isocyanate, assuming that all of the sample extracted was isocyanates). Then, 970 μL of this derivatization solution was placed in a 1.5 mL screw-cap vial. Once the reaction was carried out, 30 μL of the autoclave solution was extracted and immediately mixed in the vials where the PP solution was present. DMSO was used to help homogenize the mixtures.

#### 2.2.2. Liquid Chromatography

Liquid chromatography (LC) was used to quantify the amounts of MPC, phenyl isocyanate (as PP-derivative), DPU, and aniline in the reaction mixture. LC-UV was performed on ThermoFisher Ultimate 3000 series with an Ascentis^®^ Express RP-Amide (15 cm × 2.1 mm, 2.7 μm) HPLC column accompanied by an Ascentis^®^ Express 90Å RP-Amide guard column at room temperature.

A gradient mobile phase (0.2 mL/min) consisting of (A) 5 mM ammonium acetate with deionized water (0.1%v Formic acid) and (B) Acetonitrile (0.1%v Formic acid) was used. The UV detector was set to measure at 254 nm and 310 nm. All the components are visible at 254 nm, and 310 nm is used to verify some of the components (e.g., PI). Standard solutions for the aniline, DPU, phenyl isocyanate (PI), and MPC were created with a concentration of 1 mg/mL in DMSO and then further diluted using a mixture of water: acetonitrile (1:1 v), to create calibration samples.

**Figure A3:**
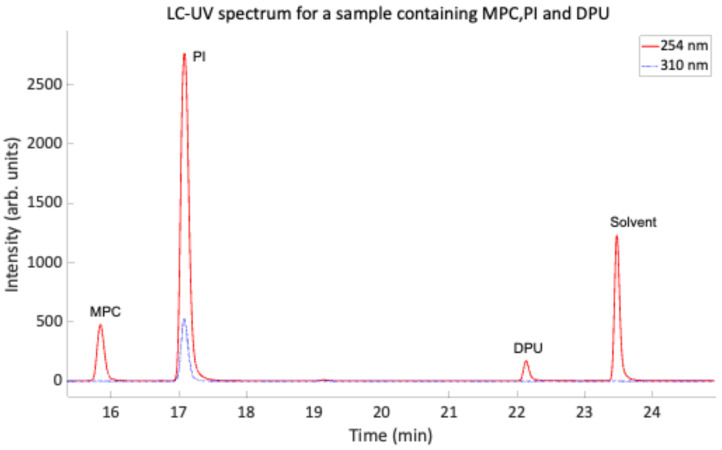
Typical LC-UV chromatogram for the expected components in the reaction mixture.

Once the extracts from the reaction were neutralized, they were prepared for analysis in LC-UV. The samples were first filtered (Whatman 0.2 μm filter) and then diluted with equal volumes of water and acetonitrile. The stability and reproducibility of the analysis conducted via LC-UV (Figure A3 have been evaluated at regular intervals to maintain a reliable method.

### 2.3. Safety Recommendations

Isocyanates are highly reactive, which could lead to severe effects on human health upon direct contact. Phenyl isocyanate, among other isocyanates and diisocyanates, is registered under the REACH regulation. Due to the isocyanates’ skin and respiratory sensitization, a low exposure limit (5 ppb for 8 h and 20 ppb for 15 min) has been assigned to workers dealing with these compounds [35,38,39,40,41,42].

The neutralization step used in the analytical method is only one step toward a safe working environment. Guidelines have been provided by occupational health and safety agencies globally [41,43,44]. Accordingly, experiments should be performed in a well-ventilated fume hood with the proper personal safety equipment as well as a neutralization agent [39] and gas detector (colorimetric tape-based instruments, impingers, etc) [43,45].

## 3. Results

The goal of this study is to obtain a good understanding of the reactions that happen in the thermal and catalytic cracking of carbamates. The main products observed in our experiments were mainly aniline (A), diphenyl urea (DPU), and phenyl isocyanates (PI). Together with the unconverted carbamate, they accounted for ≥90 mol% of the feed used for the experiments.

### 3.1. Thermal and Catalytic Carbamate Cleavage

The carbamate decomposition (MPC) was studied with and without catalysts. The aim of the non-catalytic experiments was to obtain a baseline for the decomposition of the carbamates at the reaction conditions.

**Figure 1:**
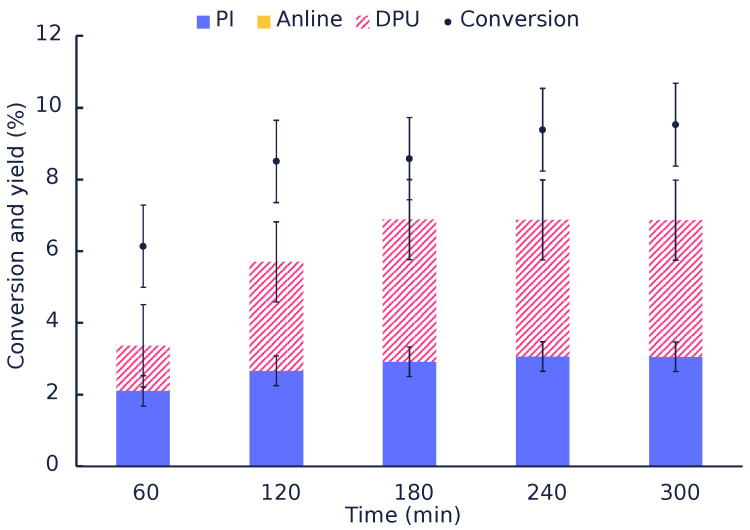
The non-catalytic conversion of MPC at 200 °C (8 wt% MPC in diphenyl ether). Negligible aniline was observed in these experiments.

MPC’s thermal (catalyst-free) cleavage was evaluated by processing MPC at 8 wt% in diphenyl ether at 200 °C and sampling the product every 60 min. According to Figure 1, MPC undergoes very slow conversion, reaching only 10% after 5 h. The products consist mainly of isocyanate (PI) and urea (DPU), with no measurable amounts of aniline and a mole balance of ∼98%.

Different catalysts that are reported in the literature were evaluated, namely zinc oxide (ZnO, 10–25 m^2^/g), aluminium oxide (Al_2_O_3_, 155 m^2^/g), montmorillonite K-10 (H_2_Al_2_(SiO_3_)_4_ −nH_2_O, 220–270 m^2^/g, and Bismuth(III) oxide (Bi_2_O_3_, 3.8–5.5 m^2^/g) [24,25,46].

A starting concentration of 8 wt% MPC in diphenyl ether and a final reaction temperature of 200 °C were selected. The catalyst-to-carbamate mass ratio is generally 6:10, except for Al_2_O_3_, where a 1:1 ratio was also tested.

**Figure 2:**
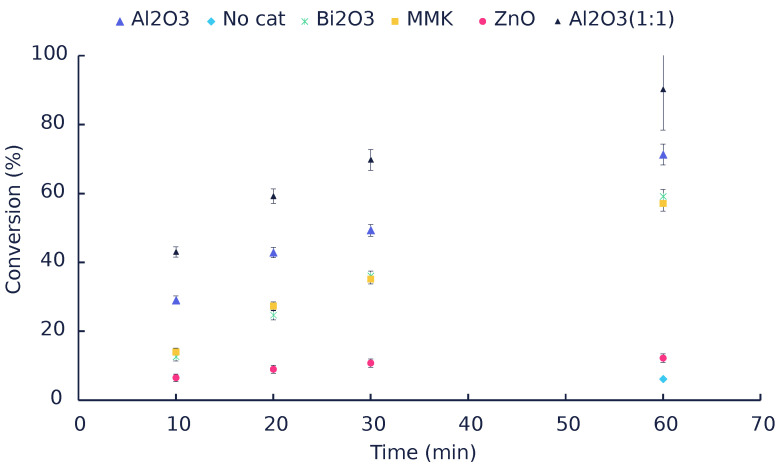
The catalytic performance of ZnO, Al_2_O_3_, Bi_2_O_3_, and MMK vs. time. (the catalyst to feed ratio is 6:10 unless stated otherwise).

Figure 2 shows that the conversion increases with time and with an increase in the amounts of catalysts used. Zinc oxide performed poorly (∼12% conversion after 1 h) compared to other catalysts. Table 1 summarizes the main product’s yields and the reaction mole balance based on aniline, DPU, PI, and MPC. In all catalytic tests performed, and the yield to PI was less than 1% or negligible, while aniline was the main compound identified. DPU also seemed to be the other major product in these experiments. In an attempt to fill the gap generally observed in the literature, mole balances were calculated based on quantified components, i.e., the isocyanate (PI), DPU, and aniline, as well as the unconverted carbamate. Therefore, any missing mole balance is due to experimental error and/or the products that we could not identify. Attempts to identify them will be discussed below.

**Figure 3:**
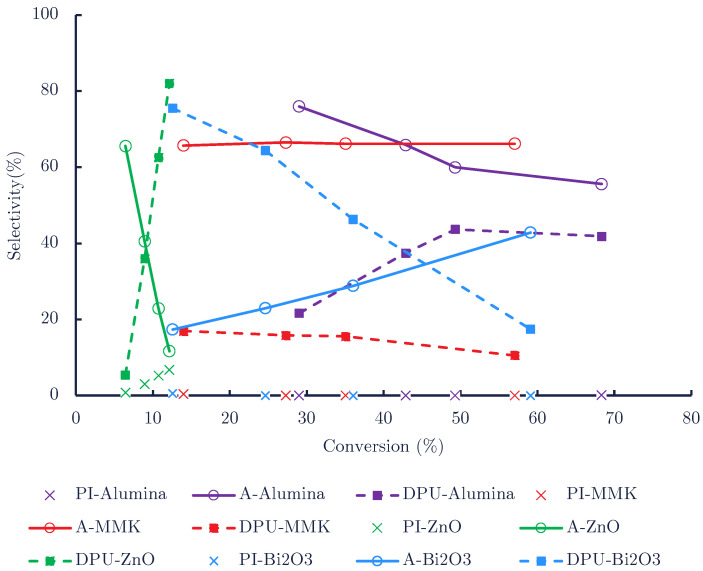
Selectivity vs. conversion in the catalytic tests with MMK, ZnO, Al_2_O_3_ and Bi_2_O_3_.

Figure 3 indicates that each catalyst showed a different profile. The most active one, Al_2_O_3_, demonstrated high selectivity to aniline (∼76 mol%) that decreases to the benefit of DPU at a high conversion. In contrast, MMK demonstrated a high and stable aniline selectivity (∼65 mol%) that parallels a much lower selectivity for DPU (∼15 mol%). Finally, the least active catalyst, ZnO, demonstrated a high selectivity of aniline that dropped very rapidly to the benefit of DPU and showed a modest production of isocyanate PI that resembled but did not exceed the one observed in the thermal, non-catalytic run.

**Figure 4:**
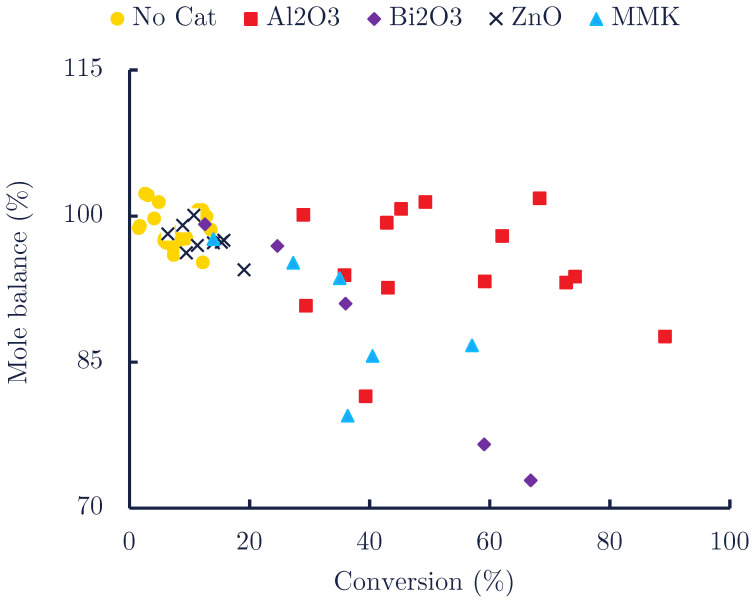
Aromatic mole balance of the quantifiable compounds vs. MPC conversion in the autoclaves (variable cat: feed ratio with a set temperature of 200 °C ).

These three products, aniline, DPU and the traces of PI, when observed, accounted for most of the converted MPC. However, minor additional products seem still to form at high conversion, as can be witnessed by the slight but significant drop in mole balance from ∼100 to ∼90 mol% for Al_2_O_3_ and ∼80% for MMK and Bi_2_O_3_, as the conversion increases to 100 mol% (Figure 4). These unidentified by-products could consist of heavy products made by the slow condensation of DPU or rapid condensation of PI with themselves, one another or with unconverted MPC, some being illustrated in Scheme 2d–g.

The absence of aniline formation in the thermal run and its dominance in the catalytic runs suggested that water had been inadvertently added to the system together with the catalysts, despite the fact that every practical measure was taken to ensure that no water content was present in the system.

#### Catalyst to Feed Ratio

To further test the hypothesis that water was being introduced into the system via the catalysts, a series of experiments was performed in which the catalyst: MPC ratio was changed. A solution of 8 wt% MPC in diphenyl ether was used in these experiments.

**Figure 5:**
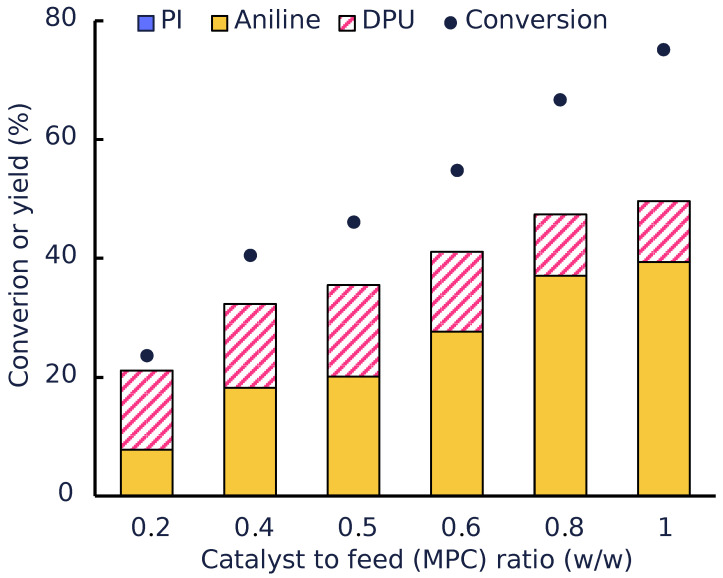
Conversion and yield of products obtained in the autoclave tests conducted with MMK-10 as the catalyst (8 wt% MPC in Diphenyl ether at 200 °C and 1 h).

**Figure A4:**
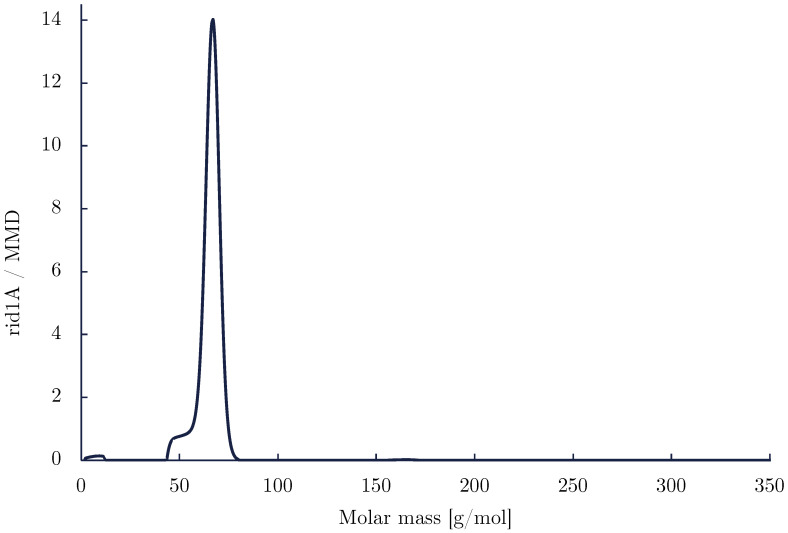
GPC chromatogram obtained from the neutralized samples, indicated too much dilution.

Figure 5 shows that the conversion of the carbamates increases as a higher amount of the catalysts is used; however, the formed products consist mainly of aniline and unidentified products, as evident by the gap between the sum of yields and the conversion. However, the GPC analysis (Figure A4) of the liquid product did not reveal the presence of heavy products. This suggests that the heavy products are mainly trapped on the catalyst surface.

### 3.2. Effect of Water

To confirm the critical role of water, a new set of experiments was performed, in which 1 g (∼6.6 mmol) of MPC was added to 10 mL glass autoclaves that were chosen, as they could withstand higher steam pressures. Afterwards, 2.7 mmol water was added to one of the autoclaves to compare the difference between pure carbamate vs. water-added carbamate decomposition.

These tests were done under the same condition and time to reduce the effect of external factors on the experiments. After 1 h at 200 °C, the autoclaves were removed from the heating block, and the sample was extracted and neutralized.

**Figure 6:**
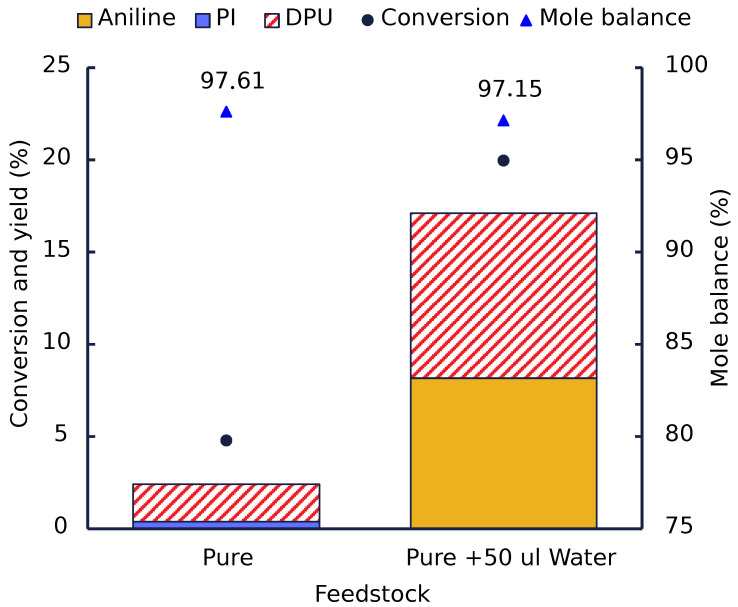
Effect of water addition (stoichiometric amount) in the thermal cleavage of MPC.

Figure 6 shows that adding water severely increased the conversion of MPC to aniline and DPU. The addition of water, even below the stoichiometric ratio, resulted in a significant conversion (∼20%) compared to dry carbamates (5%), with a substantial amount of aniline formed as a product.

It should be noticed that the aniline/DPU ratio of about 1:1 observed upon the water addition is much lower than that observed in the presence of Al_2_O_3_ or MMK catalyst at similar conversion. This suggests that the catalysts accelerate the formation of aniline (MPC hydrolysis) more efficiently than the formation of DPU (MPC aminolysis).

## 4. Discussion

Figure 1 proves that carbamate can be thermally cracked to PI (and alcohol). The levelling off of conversion at ∼10% after 3 h suggests that the cracking is limited by a thermodynamic equilibrium at a PI/MPC ratio of ∼1/30 (i.e., K∼10) at 200 °C. Higher temperatures may be needed for higher conversion.

**Scheme 4:**
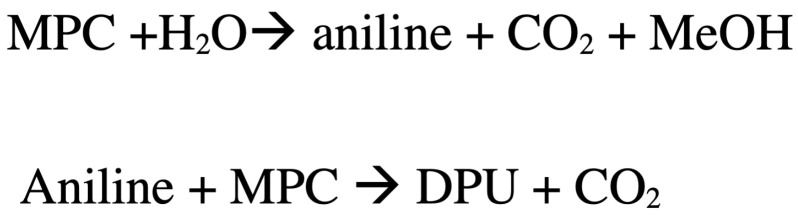
Proposed catalytic reaction for the conversion of carbamate in the presence of water.

The addition of water to the reaction mixture, either directly (Figure 6) or indirectly via the catalysts (Figure 3 and Figure 5), appears to divert the reaction from cracking to PI to MPC hydrolysis to aniline and its subsequent addition to unconverted MPC (MPC aminolysis) to form DPU. Both reactions could proceed directly from MPC or indirectly via the cracking top PI and subsequent addition of water or aniline to the PI. The indirect reaction via PI seems less likely, however. The intermediate formation of PI was not observed to any significant level, which indicates that, if proceeding, this reaction should be very slow and rate limiting. However, it then remains unclear how the addition of water would accelerate this slow cracking reaction to PI and lead to the increase in conversion that we observed. Consequently, we propose that the catalytic reaction proceeds via direct hydrolysis and aminolysis of MPC Scheme 4.

Intriguingly, the formation of DPU without intermediate observation of aniline in the purely thermal reactions remains puzzling. Formally, the net conversion of MPC to DPU requires the release of ${\mathrm{CO}}_{2}$ + dimethyl ether or dimethyl carbonate, which could not be detected in the liquid or gas cap. The most credible explanation is to assume that traces of water dissolved in the chemicals or adsorbed on the hardware would form some aniline, which directly reacts with PI according to reaction c of Scheme 2).

Further analysis of water content and the investigation of the relation between the surface structure and of the catalysts is beyond the scope of this paper. However, the catalysts play a more prominent role than only supplying water since (1) we do not observe a specific relation between the catalyst’s surface area and the conversion (for example Bi_2_O_3_ with a lower surface area to ZnO, provided higher conversion under same conditions) and (2) we find different aniline/DPU ratios at a similar conversion for the various catalysts, i.e., 6.2 for MMK, 2.4 for Bi_2_O_3_ and 1.4 for Al_2_O_3_ at about 50% MPC conversion (Figure 1).

## 5. Conclusions

Analysis of isocyanates and additional products formed during thermal and catalytical cracking of MPC proved to be challenging and critical at the same time. This could explain the lack of mole or mass balances reported in the literature. A robust and safe analytical method for quantifying isocyanates has been developed based on liquid chromatography.

Purely thermal decomposition of MPC at 200 °C successfully provided small amounts of isocyanates, which then reacted rapidly with the aniline formed by hydrolysis of MPC with residual water. However, the thermal decomposition is slow at such low temperatures and could be bound to the equilibrium limitations.

Adding catalysts promoted the formation of aniline and diphenyl urea (DPU), arguably by adding adsorbed water in the reaction medium and, thereby, promoting the MPC hydrolysis and aminolysis reactions. Beyond that, the different catalysts seem to accelerate these two reactions to different extents, as indicated by their difference in selectivity between aniline and DPU.

## Figures and Tables

**Table 1:** Conversion, products yield, and mole balances obtained in the catalytic degradation of MPC (Conditions: 200 °C and catalyst: MPC = 6:10).

Catalyst	Time (min)	Conversion (mol%)	${\mathrm{Yield}}_{\mathrm{Aniline}}\;\left(\%\right)$	${\mathrm{Yield}}_{\mathrm{PI}}\;\left(\%\right)$	${\mathrm{Yield}}_{\mathrm{DPU}}\;\left(\%\right)$	Mole Balance (%)
ZnO	10	6.5	4.2	0.1	0.3	98.2
	20	8.9	3.6	0.3	3.2	98.2
	30	10.7	2.5	0.6	6.7	99.0
	60	12.2	1.4	0.8	10.0	100.1
MMK	10	14.0	9.2	0.1	2.4	97.6
	20	27.3	18.1	0.0	4.3	95.2
	30	35.1	23.2	0.0	5.4	93.6
	60	57.0	37.7	0.0	6.0	86.7
Bi_2_O_3_	10	12.6	2.2	0.1	9.5	99.2
	20	24.6	5.7	0.0	15.9	96.9
	30	36.1	10.4	0.0	16.7	91.0
	60	59.1	25.3	0.0	10.3	76.5
Al_2_O_3_	10	29.0	22.0	0.0	6.3	99.3
	20	42.9	28.2	0.0	16.1	101.4
	30	49.3	29.5	0.0	21.5	101.8
	60	68.3	38.0	0.1	28.6	98.3

**Figure A5:**
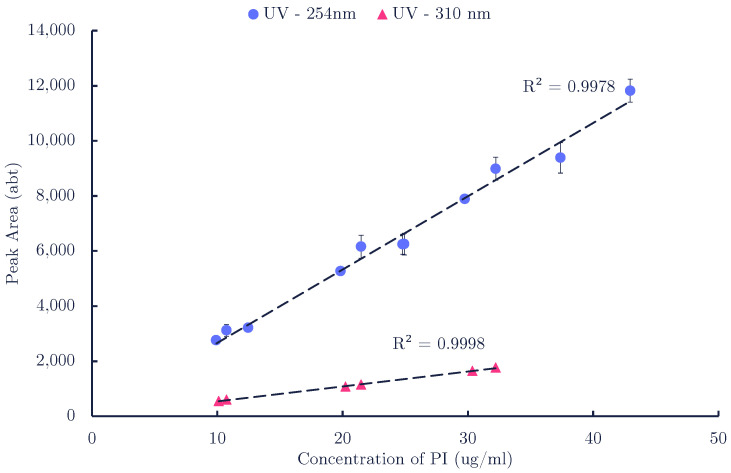
LC-UV Calibration curve for PI at 254 nm and 310 nm wavelength.

## Data Availability

All data obtained within this work is available at request and are to be stored within the University of Twente data archive for a minimum of 10 years.

## Abbreviations

The following abbreviations are used in this manuscript:

MPC	Methyl N-phenyl carbamate
PUR	Polyurethanes
TDI	Toluene diisocyanate
MDI	Methyl diphenyl diisocyanate
HDI	1,6-hexameylene diisocyanate
DPU	1,3-Diphenylurea
MMK	Montmorillonite K-10
HPLC	High performance liquid chromatography
LC	Liquid chromatography
MS	Mass spectrometer
UV	Ultraviolet
PP	1-(2-Pyridyl)piperazine
DMSO	Dimethyl sulfoxide
PI	Phenyl isocyanate
wt%	Weight percent
GPC	Gel permeation chromatography
